# Reconstruction of a Volar Thumb Defect Using a Princeps Pollicis Artery Perforator Flap: A Case Report of Partial Flap Congestion and Successful Salvage

**DOI:** 10.7759/cureus.107003

**Published:** 2026-04-14

**Authors:** Nay Aung Zin, Thura Kyaw, Phyo Thant, Mohamed Arsham Abdul Rasheed, Ko Ko Kyaw, Phyo Aung Kyaw

**Affiliations:** 1 Orthopaedics and Traumatology, Kulhudhuffushi Regional Hospital, Kulhudhuffushi, MDV; 2 Orthopaedics and Trauma, 300 Bedded Orthopaedic Hospital, Mandalay, MMR; 3 Orthopaedic Surgery, University of Medicine 1 Yangon, Yangon, MMR; 4 Orthopaedic Surgery, Kulhudhuffushi Regional Hospital, Kulhudhuffushi, MDV; 5 Emergency, Indira Gandhi Memorial Hospital, Malé, MDV; 6 Intensive Care Unit, Indira Gandhi Memorial Hospital, Malé, MDV

**Keywords:** electrical injury, hand surgery, local flap, perforator flap, princeps pollicis artery perforator flap, soft tissue reconstruction, thumb reconstruction, volar thumb defect

## Abstract

Reconstruction of volar thumb defects remains challenging due to the requirement for durable, sensate, and glabrous tissue. Local perforator-based flaps have emerged as reliable options that preserve thumb length and function.

We present a case of a 21-year-old female with a volar soft tissue defect over the proximal phalanx of the right thumb following electrical injury. Initial debridement was performed on the first day post-injury, followed by reconstruction using a princeps pollicis artery perforator flap on day 7. The flap measured 2.8 × 2.3 cm and was elevated under brachial plexus block anesthesia. Postoperatively, the patient developed venous congestion between days 3 and 7, which was managed conservatively with limb elevation, partial suture release, and culture-directed antibiotic therapy. The flap subsequently stabilized, with satisfactory healing. At six months’ follow-up, the patient demonstrated full opposition to all fingers, a power grip of 250 N, and key pinch strength of 5 kg, with the ability to perform daily activities. Mild extension lag of 15 degrees and scarring were noted as residual drawbacks.

This case highlights the utility of the princeps pollicis artery perforator flap as a single-stage, function-preserving reconstructive option for volar thumb defects, even in the presence of postoperative complications.

## Introduction

Electrical injuries to the hand are relatively uncommon but can result in significant soft tissue damage, neurovascular compromise, and long-term functional impairment. Due to the high resistance of skin, electrical burns often produce deep tissue necrosis that is disproportionate to the apparent surface injury, frequently extending beyond clinically visible margins [1].

The thumb plays a critical role in hand function, contributing approximately 40%-50% of grip and pinch strength and is essential for opposition and fine motor tasks [2]. Therefore, restoration of thumb integrity, along with functional and aesthetic outcomes, is a primary goal in reconstructive surgery. Reconstruction of thumb defects is particularly challenging due to the need for durable, sensate coverage while preserving mobility and strength.

Various reconstructive options have been described, including skin grafts, local flaps, regional flaps, and free tissue transfer [3]. However, skin grafts may be insufficient in cases with exposed vital structures, while more complex procedures, such as free flaps, require microsurgical expertise and are associated with increased operative time and morbidity.

Local perforator-based flaps have emerged as a reliable alternative, utilizing adjacent tissue with preserved vascularity while avoiding the sacrifice of major vessels. These flaps offer several advantages, including minimal donor-site morbidity and excellent color match and textural consistency with the surrounding native tissue [4]. The vascular supply of the thumb is primarily derived from branches of the radial artery, including the princeps pollicis artery, which provides a consistent perforator suitable for flap design.

Perforator flaps based on the princeps pollicis artery represent a dependable option for thumb reconstruction, allowing adequate coverage with favorable functional and aesthetic outcomes [5].

This report presents a case of volar thumb reconstruction following electrical injury using a perforator-based flap, highlighting surgical planning, intraoperative technique, postoperative management, and functional recovery.

## Case presentation

**Figure 1:**
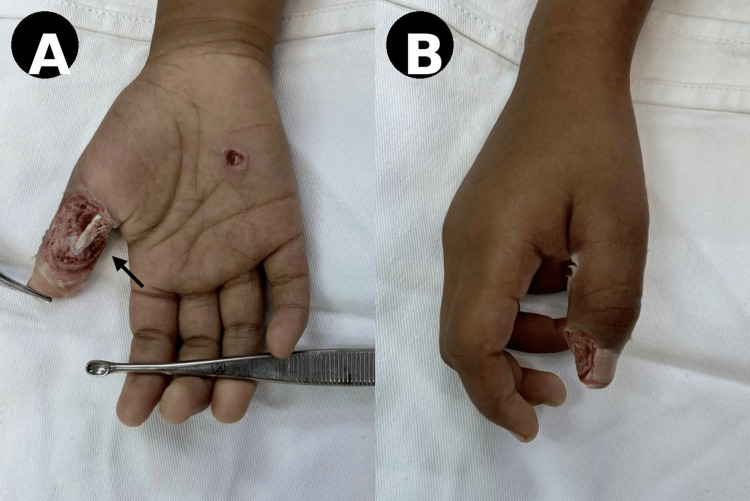
Initial presentation (A) Volar view showing a full-thickness soft tissue defect with exposed structures (arrow). (B) Lateral view demonstrating the extent of tissue loss and thumb deformity.

A 21-year-old, right-hand-dominant female presented with an electrical burn injury involving the volar aspect of the proximal phalanx of the right thumb. Clinical examination revealed a full-thickness soft tissue defect with exposed underlying structures, along with a small punctate wound over the palm (Figure 1). The injury resulted in significant tissue loss and functional limitation

**Figure 2:**
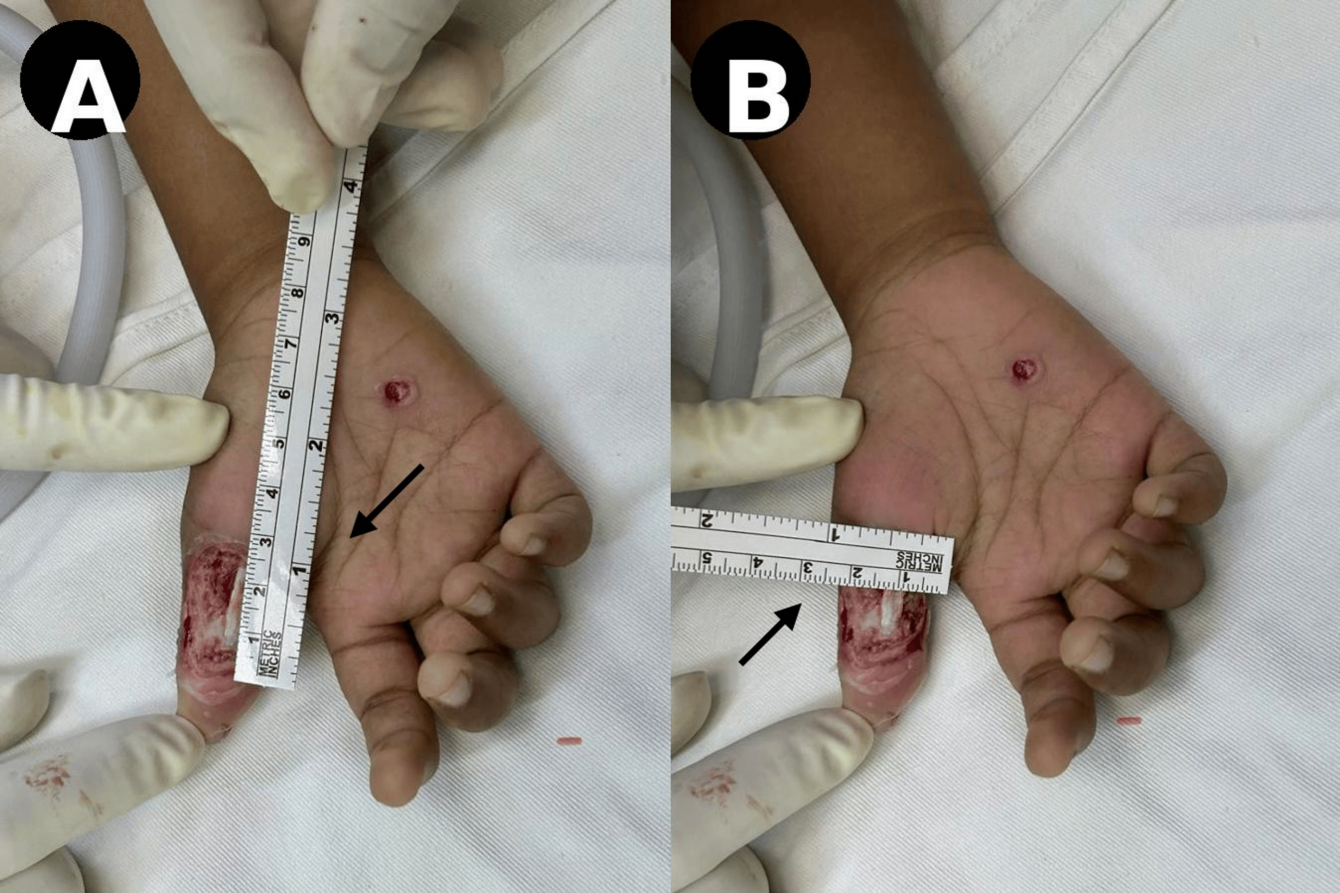
Defect assessment and measurement (A) Measurement of defect length on the volar thumb (arrow). (B) Measurement of defect width confirming size and extent (arrow).

Initial surgical debridement was performed on the first day following injury to remove devitalized tissue and reduce the risk of infection. Subsequent assessment of the defect included precise measurement of its dimensions and evaluation of surrounding tissue viability (Figure 2).

**Figure 3:**
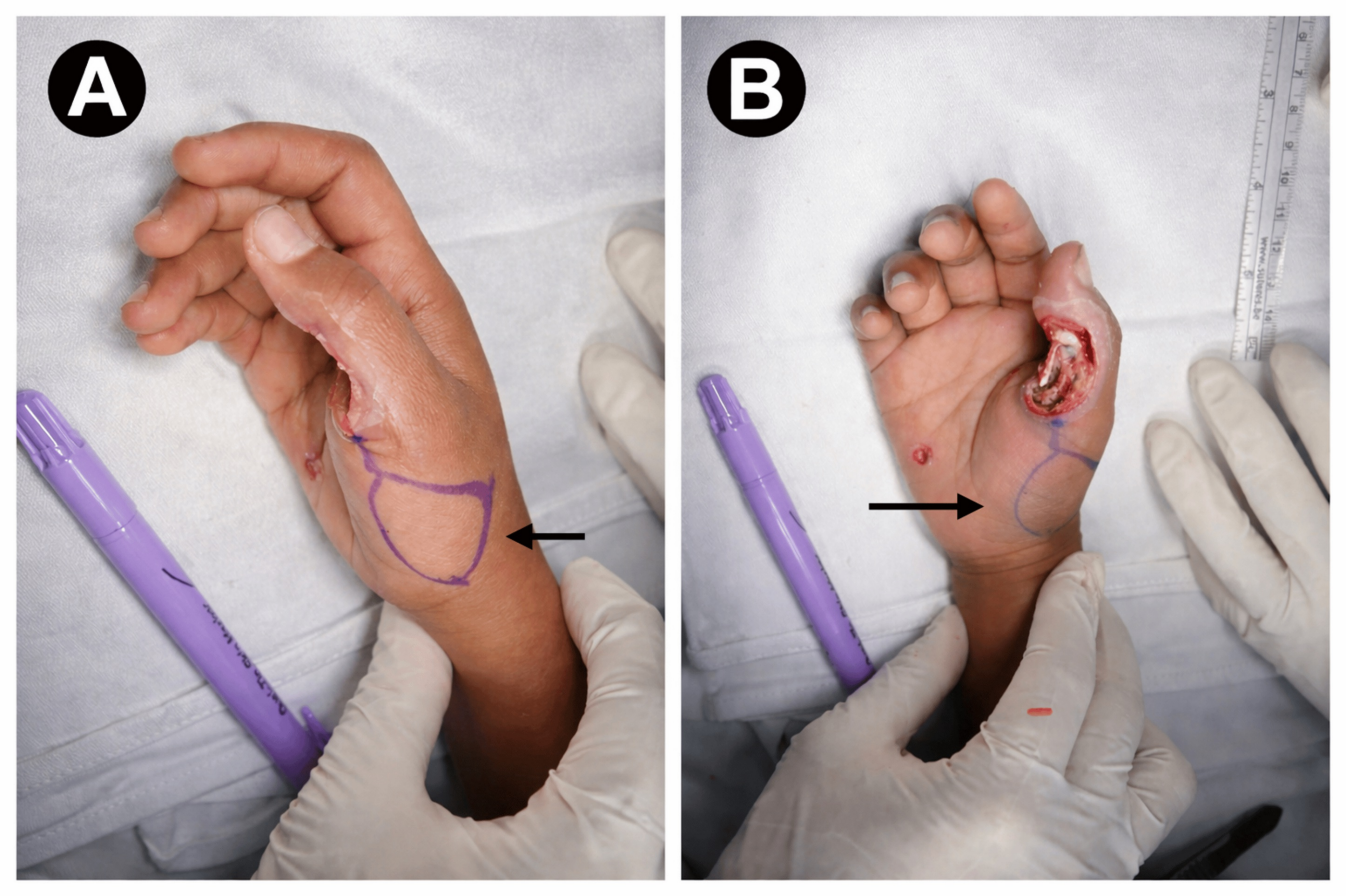
Preoperative flap planning (A) Marking of the perforator-based flap over the radial mid-palmar region (arrow). (B) Correlation of flap design with recipient defect (arrow).

Definitive reconstruction was planned using a perforator-based local flap. Preoperative markings were made over the radial mid-palmar region based on the princeps pollicis artery perforator (Figure 3).

**Figure 4:**
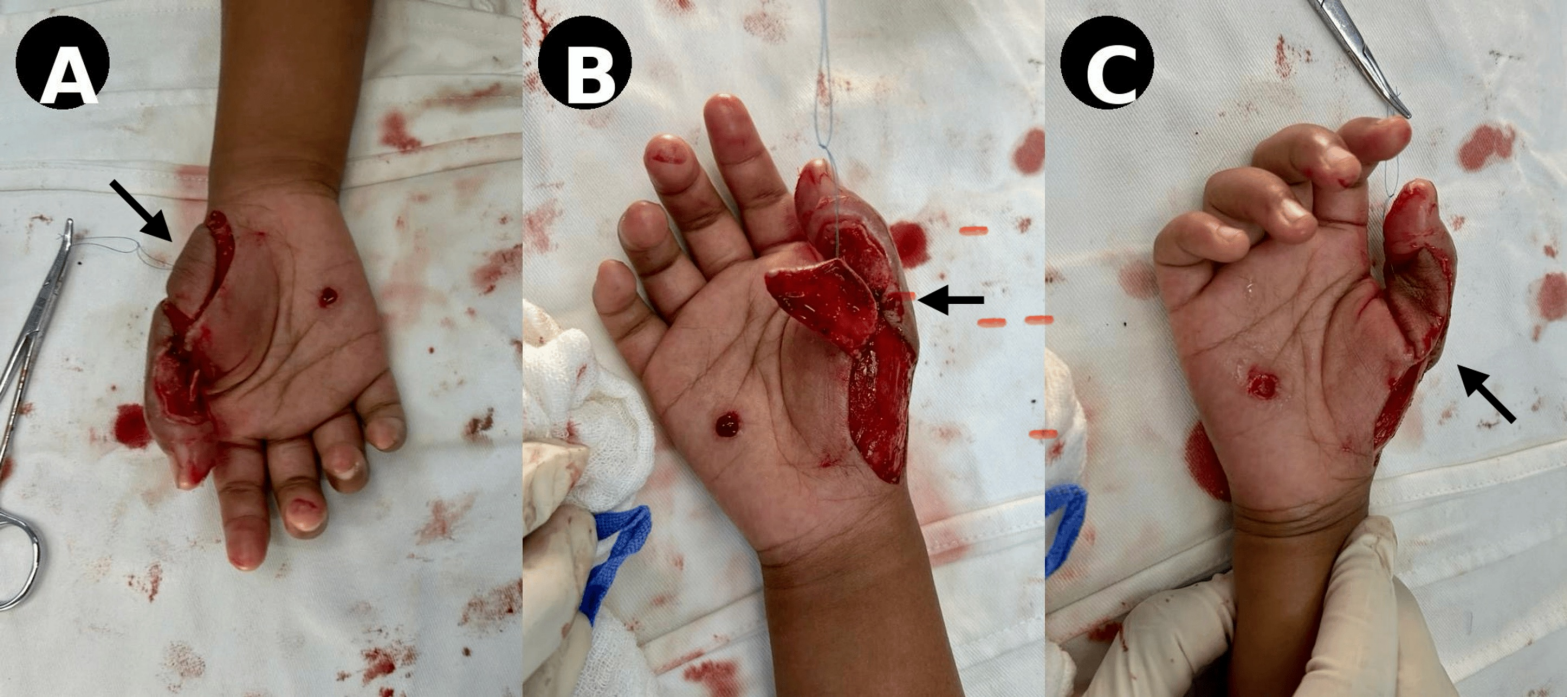
Intraoperative findings (A) Post-debridement defect with exposed structures (arrow). (B) Flap elevation demonstrating vascularized tissue (arrow). (C) Flap rotation toward defect (arrow).

On postoperative day 7, reconstruction was performed under brachial plexus block anesthesia. Following adequate debridement, the defect was reassessed (Figure 4A). A flap measuring approximately 2.8 × 2.3 cm was elevated with careful identification and preservation of the perforator vessel arising from the superficial palmar branch of the radial artery (Figure 4B). The flap was then rotated and inset into the thumb defect without tension (Figure 4C).

**Figure 5:**
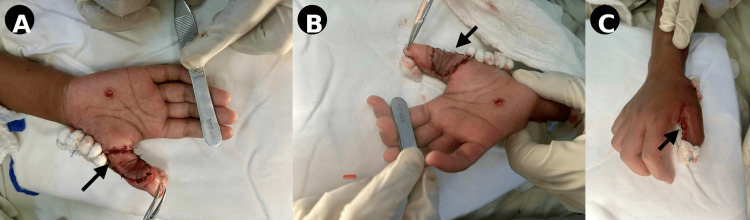
Immediate postoperative period (A and B) Early postoperative appearance with flap in situ (arrow). (C) Alternative view showing donor site and flap coverage (arrow).

The flap was secured in place with satisfactory initial perfusion, and the donor site was managed with partial closure and split-thickness skin grafting (Figure 5).

Immediate postoperative assessment showed a viable flap with good coverage of the defect.

**Figure 6:**
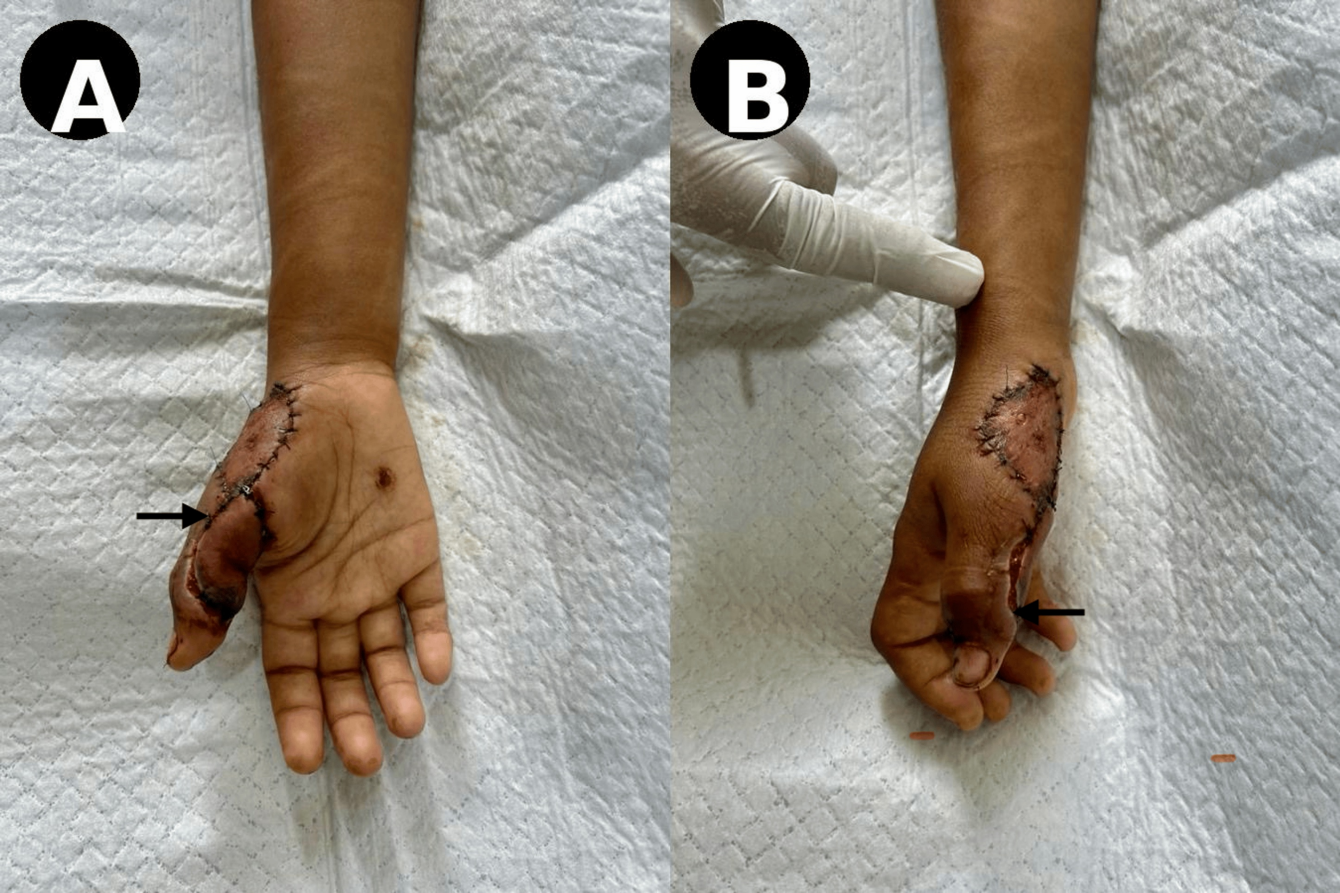
Early complications (Post-op days 3-10) (A) Venous congestion with discoloration of the flap (arrow). (B) Progression of congestion and edema (arrow).

During the early postoperative period, between postoperative days 3 and 10, the flap developed venous congestion characterized by discoloration and edema (Figure 6).

Conservative management was initiated, including limb elevation and partial suture release to relieve venous outflow obstruction. Culture and sensitivity testing guided antibiotic therapy, and the patient was treated with oral levofloxacin 500 mg once daily for 10 days. These measures resulted in a gradual improvement in flap vascularity, and further necrosis was prevented.

**Figure 7:**
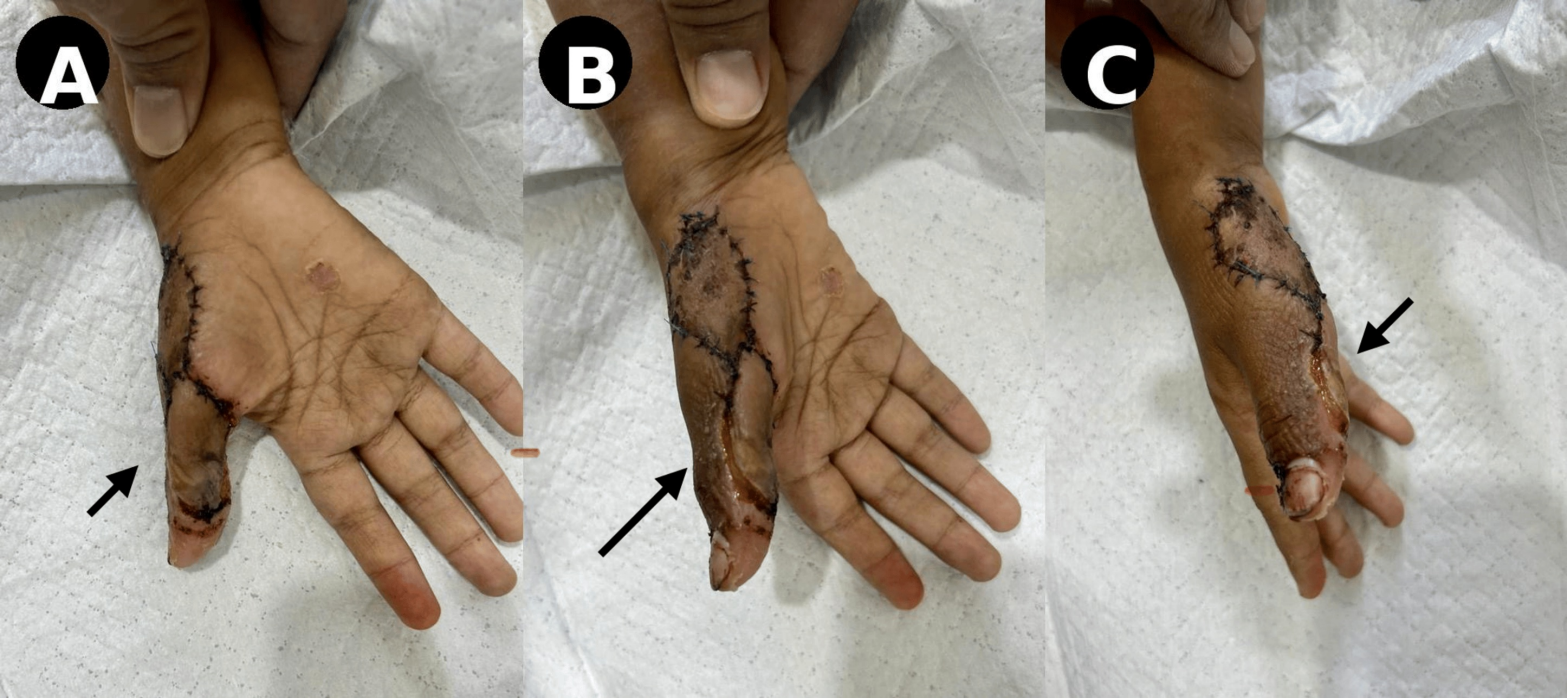
Intermediate follow-up (six weeks) (A) Healing flap with improved vascularity and contour. (B and C) Residual scarring and ongoing remodeling.

At the six-week follow-up, the flap demonstrated progressive healing with acceptable contour and improved tissue quality (Figure 7).

**Figure 8:**
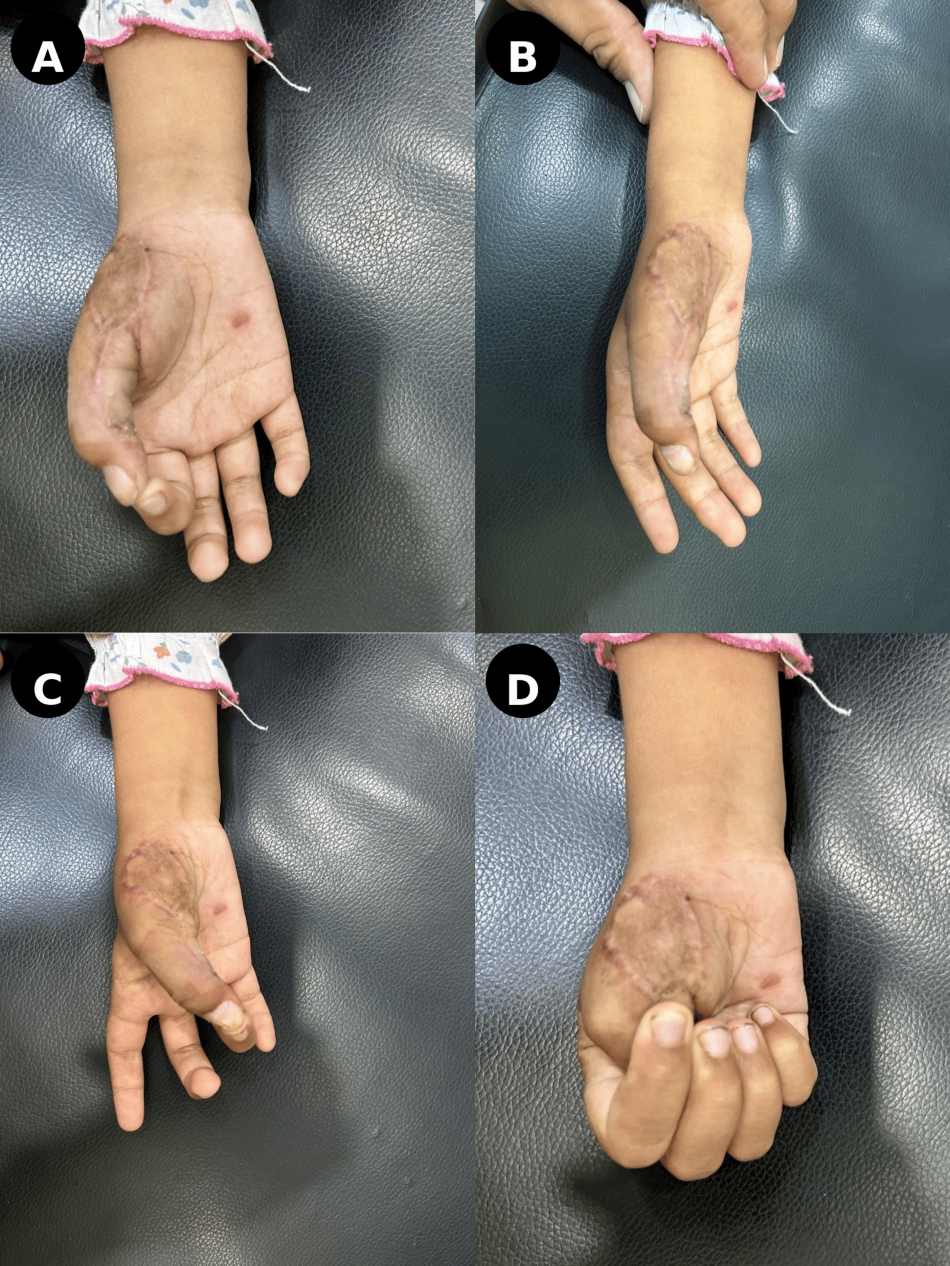
Final outcome (six months) (A and B) Well-healed flap with stable soft tissue coverage. (C and D) Functional outcome demonstrating thumb opposition and grip.

At final follow-up at six months, the flap had healed completely, providing stable soft tissue coverage (Figure 8).

The patient achieved full opposition of the thumb to all fingers, with a power grip strength of 250 N and key pinch strength of 5 kg. A mild extension lag of approximately 15 degrees and minimal scarring were noted but did not significantly impair function, and the patient was able to perform daily activities independently.

## Discussion

Electrical injuries are characterized by unpredictable tissue damage due to thermal effects and vascular compromise, often resulting in progressive necrosis beyond the visible wound margins [1]. Early debridement is therefore essential to remove devitalized tissue and reduce infection risk while preparing the wound bed for reconstruction [6].

Reconstruction of thumb defects remains challenging because of the need to restore both durable coverage and functional mobility. Given the thumb’s critical role in hand biomechanics, reconstructive techniques must prioritize preservation of opposition, grip strength, and sensibility [2].

In this case, the flap provided stable soft tissue coverage; however, venous congestion developed in the early postoperative period, which is a recognized complication of perforator-based flaps due to relatively delicate venous drainage [6]. Conservative measures, including limb elevation, partial suture release, and continuation of prophylactic antibiotics to minimize infection risk, were effective in restoring venous outflow and preventing flap compromise [6,7].

At six months, the patient demonstrated satisfactory functional recovery, including restoration of thumb opposition, adequate grip strength, and the ability to perform age-appropriate daily activities. Although a formal validated scoring system was not applied, clinical assessment showed meaningful functional improvement consistent with reported outcomes in similar reconstructions [4,5]. Importantly, mild extension lag and minimal scarring were observed but did not significantly impact overall hand function [4].

From an aesthetic perspective, the flap demonstrated excellent color match and textural consistency with the surrounding native tissue, supporting the suitability of perforator-based flaps in pediatric thumb reconstruction. These findings align with previous studies reporting favorable functional and aesthetic outcomes, low donor-site morbidity, and reliable vascularity with such techniques [3-5].

## Conclusions

The princeps pollicis artery perforator flap is a reliable and effective option for the reconstruction of volar thumb defects. It enables single-stage reconstruction using glabrous tissue while preserving thumb integrity, function, and aesthetics, with minimal donor-site morbidity. Even in the presence of postoperative complications such as venous congestion, satisfactory functional and aesthetic outcomes can be achieved with timely and appropriate management. The flap also demonstrates excellent color match and textural consistency with the surrounding native tissue, supporting its utility in hand reconstruction.
